# Exploring relationships between college students’ social networks, social support, and mental health during the COVID-19 pandemic

**DOI:** 10.1371/journal.pmen.0000289

**Published:** 2026-03-20

**Authors:** Michael D. Broda, Tzu-Wei Wang, John Hui, Erica Ross, Hollee A. McGinnis, David M. Chan, Camie A. Tomlinson, Claire Luce

**Affiliations:** 1 Department of Foundations of Education, Virginia Commonwealth University, Richmond, Virginia, United States of America; 2 School of Social Work, Virginia Commonwealth University, Richmond, Virginia, United States of America; 3 Department of Mathematics and Applied Mathematics, Virginia Commonwealth University, Richmond, Virginia, United States of America; 4 Kent School of Social Work and Family Science, University of Louisville, Louisville, Kentucky, United States of America; University of Science and Technology of China, CHINA

## Abstract

This study investigates the relationship between college students’ social networks, social support, and mental health in the midst of the COVID-19 pandemic. Using data from a large public research university, the research explores the associations between perceived social support from friends, family, and significant others, social network characteristics, and their individual and combined association with mental distress. By applying Social Capital Theory and Relational Regulation Theory, the study highlights the critical role of social interactions and support systems in mitigating mental health challenges during the pandemic. The findings underscore the importance of family support and high-quality social networks in reducing mental distress.

## Introduction

The COVID-19 pandemic ushered in an era of US higher education that had not been observed for more than a century. Beginning in March 2020, as the coronavirus spread widely across the US, colleges and universities rushed to contain its potential impact on students, staff, and faculty. Classes across the country were canceled for weeks, and students were sent home and asked to complete the semester online. Many observers have noted that the higher education system, both in the US and around the world, may have been permanently altered by the pandemic and these sudden changes [1–3].

It is difficult to overstate the impact of the pandemic and the resulting systemic changes to higher education on postsecondary students. Even before the onset, college students experienced a well-documented increase in mental health issues, including increased depression, stress, and anxiety [4–6]. Then, in a matter of days, campuses were closed and students were sent home, causing a significant disruption to their school-based friendship and support networks. This raised legitimate concerns about the potential deepening of the mental health crisis in US college students [7,8], as well as the potential for the pandemic to function as a significant traumatic experience [9,10].

Amid this rapid transition and disruption in March 2020, our team began collecting survey data on undergraduate and graduate students’ mental health, academic performance, and social network ties at a large, comprehensive, public research university located in the mid-Atlantic US. Our goal was to offer a unique perspective on how students’ experiences and attitudes, as well as their support networks, may have been impacted by the pandemic, focusing on how this may relate to issues of mental health. Hence, this study sought to understand the effects of the pandemic and correlations between student social support, support networks, and mental health. In addition, we used the lens of Relational Regulation Theory [11] to offer new insight into the complex interplay between these dimensions.

## Theoretical foundations

We situate the current study primarily in both Social Capital Theory and Relational Regulation Theory. Social capital is complex, and can be conceptualized differently depending on the discipline, framework, and level of investigation [12,13]. Despite this lack of a single definition, there is a general consensus that social capital cannot exist without network connections [14–16]. There is also consensus regarding the components of social capital. Two types of social capital have been identified: bonding and bridging [17,18]. Bonding social capital is exclusive and refers to the relationship between individuals in homogeneous groups or similar others. Examples of bonding social capital include sports teams in college, family members, close friends who one would approach first when seeking help, or members of the same church or social club. By contrast, bridging social capital is inclusive and focuses on the relationship between diverse groups. Members might share the same purpose, yet they have various backgrounds compared to bonding. Examples of bridging include student advocacy groups, student governments in colleges, and youth service groups [17–19].

Researchers focusing on social capital and mental health have explored both the macro and micro levels as well as bonding and bridging [19,20]. Previous research has found that the presence of high levels of bonding social capital positively affected mental health [19]; therefore, we explored whether it also positively affects mental health during the COVID-19 pandemic.

This study focused on bonding, exploring the structural aspects (specifically *social networks*) and the cognitive aspects (specifically *perceived social support*) of individuals in relation to their bonding-type social groups or significant others. This study also aligns with Lin’s conceptualization that social capital occurs from “investment in social relationships with expected returns” (p. 30) [21]. In other words, in order to invest in your own social capital, one must interact and communicate with others.

### Relational regulation theory

The Relational Regulation Theory (RRT) was specifically developed to better understand the relationship between social support and mental health. Lakey and Orehek first introduced the theory in their conceptual review by offering a framework to explain the main effects of perceived social support and mental health [11]. They frame the theory as “how perceived support and mental health are rooted in social interaction and should clearly distinguish between social and personality influences.” [11] RRT acknowledges that perceived social support is linked to mental health, with the additional hypothesis that main effects occur when people regulate their thoughts and actions through ordinary conversations and shared activities, not just through intentional conversations about how to cope with stress [11]. That said, perceived social support is generated from “day-to-day” interactions, such as daily conversation, and casual activities like watching TV and listening to music (p. 487) [11]. A central tenet of the theory is that social support is a relational construct in which individuals determine what interaction they deem to be supportive.

This theory also aligns with the idea that the wider the availability of relationships, the greater the opportunity for effective regulation. In other words, people can regulate their affect to improve their mental health by being involved in a variety of different social relationships. To better investigate the main effects between perceived social support and mental health, Lakey and Orehek argued that instead of asking respondents about their sense of perceived support in general, investigators should ask recipients to “rate a single, most important support provider” [11]. By doing so, the investigators could minimize the bias of recipients’ influences by averaging across multiple providers.

Incorporating both Social Capital and Relational Regulation Theory allows this study to emphasize the importance of resources and connections when considering the main effect between perceived social support and mental health. RRT reveals the importance of maintaining regular social interactions in our networks to maintain mental health, rather than focusing entirely on enacted support. It also emphasizes the recipient’s perceived support by stressing the importance of determining what communication or activities recipients deem supportive [11,22]. Both social capital and RRT support the idea that when a participant has more connections and resources, including supportive communication, they will experience reduced stress and improved mental health.

### Social support among college students

Social support is a key protective factor for mental health and typical academic challenges among college students. Higher levels of perceived social support have been associated with lower levels of depression and anxiety [23,24]. Further, social support can enhance students’ ability to navigate and adjust to the challenges of college life [22,25,26] and promote educational achievement [27,28]. Researchers found that the existence of strong social support can reduce the threat to one’s well-being when encountering stressful situations, such as those that college students deal with in academic life and the adjustment process to college [29,30]. Stressful situations can also encompass unpredictable events, such as pandemics. During the pandemic, college students faced a radical disruption of their daily lives, including campus lockdown, quarantine, and remote learning. Researchers examining the pandemic have found social support to be a consistent protective factor against stress among college students [22,31,32].

### Personal networks

Social ties are a mechanism within the larger field of social network analysis that measures individual social capital. Social capital has both structural and cognitive components [17]. The structural component refers to relationships and networks that connect people. Cognitive components refer to the reciprocity and values inherent in those connections, such as shared values or trust [17]. For example, trust is a cognitive component that has been studied in relation to mental health, and research has shown that individual-level trust in others is inversely correlated with mental illness [33].

Social network analysis is a useful framework in this study given that social support is enacted within social networks [34]. Capturing an individual’s social network involves identifying specific actors within a person’s support network, thereby providing a more precise estimate of the sources and variability of social support relative to an aggregate measure (e.g., “on average, how supportive are your friends?”) [11].

Emerging evidence suggests that college students with stronger and closer connections to actors in their social networks have lower levels of depression and anxiety than those with weaker ties [35,36]. Studies have also suggested that larger networks function as a protective factor against depression [11,37]. In terms of the pandemic context, Elmer et al [38] found that the mandatory quarantine and the unexpected transition to online learning created dramatical changes in social interactions. Their study also showed that students were able to maintain stronger social ties when the connections were closer, whereas the COVID-19 pandemic still led to a reduction in the size of students’ overall social networks [38].

Among social network analyses, two approaches are recognized: sociometric and egocentric. Sociometric requires researchers to define the specific boundaries of the network and to obtain the complete name list of members in the networks. For example, a roster includes all the student names in a certain class. In contrast, egocentric network analysis focuses on exploring the social networks of individuals, without requiring a specific organizational boundary or complete list of members [39,40].

Consistent with Lakey and Orehek’s suggestions in RRT [11], the introduction of social network analysis in this study enabled us to transcend the limitations of assessing general perceived support by providing specific information about the support providers of college students. Furthermore, our network analysis encompasses the structural and cognitive components of social capital, which will be elaborated on in the subsequent measurement section.

## Current study

Although prior studies have examined mental health during the pandemic, the relationships between social support, social networks, and mental health have not been explored simultaneously among college students or during events as intense as the lockdowns associated with the COVID-19 pandemic.

Based on the concepts of bonding social capital and relational regulation theory, the current study used multiple approaches to examine the primary associations between perceived support, social ties, and mental health. RRT offers a valuable framework for understanding these associations. We used the MSPSS to measure perceived support from various providers (family members, friends, and significant others) among college students. Recognizing the limitations of general perceived support, as depicted in prior research [11], we employed social network analysis to delve deeper into the specific supportive social connections among college students. Moreover, social network analysis has enabled us to capture and elucidate both the structural and cognitive components of bonding social capital [17]. The current study examined these relationships and addressed the following research questions:

### Research questions and hypotheses

Three research questions and related hypotheses were addressed in our study. First, what is the relationship between perceived social support and college students’ mental distress? We hypothesized that perceived social support from various providers was negatively associated with college students’ mental distress.

H_1_: perceived social support is negatively associated with college students’ mental distress.

Second, what is the relationship between the characteristics of social networks, including their size and quality, and college students’ mental distress? We expected that social network characteristics would be negatively associated with college students’ mental distress.

H_2_: social network size and quality are both negatively associated with college students’ mental distress.

Third, combining the first two questions and hypotheses, we sought to determine whether combining perceived support and social network characteristics yields a more robust explanation of the association between social support (perceived social support and networks) and mental distress. We hypothesized that both indicators would be significantly associated with the dependent variables, and also that a model combining network and perceived support measures would explain more variability in our outcomes than a model containing only measures of perceived support.

H_3_: perceived social support and characteristics of social networks are both significantly associated with college students’ mental distress in one model.

H_3-1_: a model including both predictors would explain more variability in outcomes than those models including only one predictor.

## Method

### Data collection and sample

Cross-sectional data were collected over an eight-week period from April 7^th^ to June 2^nd^, 2020. This was approximately three weeks into the initial lockdown and campus closure. Participants were recruited via the university’s daily digital news and events newsletter, as well as via targeted emails to student email lists from major colleges and schools within the university. Participants provided written consent and received a $20 gift card as compensation for their participation in the study.

All participants were required to be enrolled at a large, research-intensive university located in the mid-Atlantic region of the United States. After completing a screening questionnaire verifying this status, participants were provided with a link to complete the online survey using REDCap (Research Electronic Data Capture, Version 4.14.5). This sample comprised *N* = 412 students (Table 1).

**Table 1:** Descriptive statistics and reliability of the IVs and DV.

Measures	*N*	*M*	*SD*	*min*	*max*	*alpha*	*omega*
Mental distress	412	0.82	0.61	0.00	2.67	0.94	0.95
Family support	412	4.73	1.30	1.50	6.50	0.83	0.89
Friends support	412	5.31	1.09	1.33	6.33	0.79	0.81
Significant other support	412	5.45	1.22	1.50	6.50	0.86	0.91
Quality of social networks	294	4.23	0.60	0.60	5.00	n/a	
Closeness of social networks	294	3.24	0.59	0.80	4.00	n/a	
Network size	412	3.77	2.48	0.00	10.00	n/a	

*Notes* According to DeVellis & Thorpe [47], Cronbach’s alpha values from.70-.80 are considered Adequate,.80-.90 are considered Very Good, and above.90 are considered excellent. Alpha values for all scales used here are adequate or above.

### Ethical approval statement

All study procedures were approved by Institutional Review Board (Protocol #HM20016878) at the authors’ university. All participants provided written consent prior to participation in the study.

### Measures

Measures of perceived social support, mental distress, and social networks were used in this study. For all measures, we calculated internal consistency using Cronbach’s alpha and McDonald’s omega. The results of our analysis are shown in Table 2, along with the relevant descriptive statistics.

**Table 2:** Descriptive statistics for final model sample.

Variables	Overall (*N* = 412)
Age (in years)	
Mean (SD)	22.61 (5.76)
Gender	
Cisgender woman	319 (77.4%)
Cisgender man	72 (17.5%)
Transgender woman	2 (0.5%)
Transgender man	4 (1.0%)
Nonbinary	9 (2.2%)
Other	6 (1.5%)
Race/Ethnicity	
Arab/Arab American	9 (2.2%)
Asian/Asian American	71 (17.2%)
Black/African American	72 (17.5%)
First Nations/Indigenous	1 (0.2%)
Latina/Latino/Latinx	36 (8.7%)
Multiracial/Mixed Race	36 (8.7%)
South Asian/Pacific Islander	16 (3.9%)
White	168 (40.8%)
Other	3 (0.7%)
Program Status	
Undergraduate	315 (76.5%)
Graduate	97 (23.5%)

#### Perceived social support.

The Multidimensional Scale of Perceived Social Support (MSPSS) is a measure of perceived social support from three sources: family, friends, and significant others [24]. The MSPSS includes 12 questions on a 7-point Likert scale ranging from 1 (“very strongly disagree”) to 7 “very strongly agree.” This measures the amount of perceived social support that the participant receives from each of the three sources. The three sources comprised three subscales with four questions for each subscale. Example items include: “*I have friends to talk to about the good and bad things in my life*” (friends), “*My family really tries to help me*” (family), and “*I have a special person who makes me feel supported*” (significant other). Scores were computed by taking the mean of relevant items to obtain each subscale score. Higher scores indicate higher levels of perceived social support. High levels of reliability and construct validity have been previously established for this measure among US college students [41,42].

#### Mental distress.

Mental distress was measured using the 21-item Depression, Anxiety, and Stress Scale (DASS-21) [43], which is a modified version of the original DASS developed by Lovibond and Lovibond [44]. This measure includes 21 items on a Likert-type scale rated 0 (“did not apply to me at all”) to 3 (“applied to me very much or most of the time”). Example items include the following: “*I found it hard to wind down*” (stress), “*I found it difficult to work up the initiative to do things*” (depression), and; “*I felt I was close to panic*” (anxiety). Participants were asked to indicate the extent to which each item applied to them over the last week. Each subscale (i.e., depression, anxiety, stress) contains seven items. In the current study, we used a total average score for mental distress. The validity and reliability of this scale have been established in previous studies using undergraduate and broader post-secondary samples [45].

#### Social network.

Social networks were assessed using compositional measures of an egocentric network analysis. Participants (egos) indicated information about their social networks through questions that analyzed their connections to others (alters). This measure specifically drew questions about the number of highly supportive ties, the closeness of ties, and the quality of ties. Participants were asked to identify the first and last initials of the most important people in their life, and to describe their relationship with each person. The options for relation included Parent, Sibling, Other Family Member, Significant Other, Friend, Roommate, and Other. A total of 1,555 nominations were made across the entire sample. Of these, 410 (26.4%) were identified as a Parent, 253 (16.3%) as Sibling, 98 (6.3%) as Other Family Member, 151 (9.7%) as Significant Other, 540 (34.7%) as Friend, 67 (4.3%) as Roommate, and 36 (2.3%) as Other. Each participant was asked to do this for up to ten people. This was followed by a series of network questions on each person. Example items from this measure include: “*How close do you consider yourself to this person?*” (closeness), “*Please rate the quality of your relationship with this person*” (quality). Each subscale was rated on a Likert-type scale. Closeness ranged from 0 (“Not at all close”) to 4 (“Very close”); Quality from 0 (“No Relationship”) to 5 (“Very Comfortable”); and Highly Supportive ties from 0 (“Not at all helpful”) to 10 (“Extremely helpful”).

Each subscale score was averaged to obtain the mean tie score. These results were interpreted as the higher the score, the stronger the closeness, quality, or support of the ties. The validity and reliability of this data collection approach were established in a prior study with postsecondary students [46]. Another measure of social networks is network size. We summed the total number of people listed by the participants as a proxy for this measure. Table 2 shows the descriptive statistics and reliability measures of the independent and dependent variables.

### Data analysis

Our analytic process included two distinct phases. First, we calculated pairwise Pearson correlations between all predictors and the outcome for a preliminary assessment of the relationship between all variables. Second, we used path analysis to evaluate three models matched to the three hypotheses stated above. All path analyses were conducted using the “lavaan” package in R [48,49]. This allowed us to obtain scores for all measures on a standardized metric as well as to include as many observations as possible by using full information maximum likelihood (FIML) method to deal with missing data. To ensure the models comply with the assumptions for regression, we tested the models for linearity, homoscedasticity, normality of residuals, multicollinearity, and regression outliers.

For any violated assumptions, we used bootstrapping to perform sensitivity analyses to ensure the results from path analysis were consistent. The assumptions of linearity and multicollinearity assumptions were met. Although the assumptions of homoscedasticity and residual normality were violated, the bootstrapping results suggested that the adjusted results were consistent with the results from the path analysis. We used Cook’s distance to identify two outlying observations. Nevertheless, the sensitivity analysis results suggested that the regression results without the identified outliers still aligned with the original results. Overall, we are confident that the path analysis results are trustworthy and consistent with robust estimation. Henceforth, we report all the results from the original path analysis.

## Results

### Sample description

Descriptive characteristics of the sample can be found in Table 2.

### Correlations

First, we estimated pairwise correlations between all independent variables and the outcome. Table 3 summarizes the results. In the following report, we used Cohen’s [50] conventions for small (0.1), medium (0.3), and large (0.5) effect sizes.

**Table 3:** Correlation matrix of key variables.

	Distress	Family	Friends	Sig_other	Quality	Closeness	Network Size
Distress	–	**-0.358**	**-0.185**	**-0.231**	**-0.203**	-0.082	0.043
Family	**-0.358**	–	**0.413**	**0.467**	**0.398**	**0.396**	-0.011
Friends	**-0.185**	**0.413**	–	**0.604**	**0.374**	**0.435**	0.065
Sig_other	**-0.231**	**0.467**	**0.604**	–	**0.414**	**0.403**	0.035
Quality	**-0.203**	**0.398**	**0.374**	**0.414**	–	**0.725**	0.043
Closeness	-0.082	**0.396**	**0.435**	**0.403**	**0.725**	–	-0.002
Network Size	0.043	-0.011	0.065	0.035	0.043	-0.002	–

*Notes. N* = 412. Correlations in bold are significant at the *p* < .05 level. Distress = composite score of mental distress. Family = perceived social support from family members. Friends = perceived social support from friends. Sig_other = perceived social support from significant others. Quality = self-reported quality of social network ties. Closeness = self-reported closeness of social network ties. Network size = numbers of members in self-listed social networks.

Most of the predictors showed negative correlation with mental distress. Only the size of the network was positively correlated with the outcome. However, the absolute values of the correlation coefficients were relatively small across all pairs, ranging from.043 to.358. Correlations between the three perceived social support predictors ranged from.413 to.604. Network quality and network closeness showed the strongest correlation (*r* = .725) among all the variables. Network size was not strongly correlated with either quality (*r* = .04) or closeness (*r* = -.002).

### Model 1 - Main effects of perceived social support

Next, we proceeded to estimate our three primary path models. To streamline reporting, we include standardized coefficients (β) in text. Unstandardized coefficients and all other estimated parameters can be found in Table 4 below. In Model 1, we investigated the main associations between perceived social support and mental distress. Among the three different sources of social support (family, friends, and significant others), only family support was statistically significant (β = -0.319, *p* < .001). Given the negative estimated relationship between family social support and mental distress, we can infer that increased family social support is associated with reduced mental distress. Model 1 explained 13.3% of the total variance in mental distress (*R*^*2*^ *=* .133).

**Table 4:** Path Analysis Results.

Predictors	*B*	95% CI for *B* [LL, UL]	*SE*	*Z*	β	*R* ^*2*^	Δ*R*^*2*^
Model 1 - PSS						0.133	
(Intercept)	1.751***	[1.452, 2.050]	0.153	11.479	2.887***		
Family	-0.149***	[-0.197, -0.100]	0.025	-6.034	-0.319***		
Friends	-0.003	[-0.067, 0.061]	0.033	-.099	-0.006		
Significant Other	-0.039	[-0.098, 0.020]	0.03	-1.307	-0.079		
Model 2 - Network Ties						0.047	
(Intercept)	1.81***	[1.061, 1.992]	0.237	6.429	2.517***		
Network Size	0.018	[-0.011, 0.048]	0.015	1.235	0.075		
Quality of Ties	-0.289***	[-0.444, -0.134]	0.079	-3.661	-0.288***		
Closeness of Ties	0.133	[-0.024, 0.291]	0.08	1.655	0.13		
Model 3 - Combined Model						0.16	0.027**
(Intercept)	1.836***	[1.393, 2.279}	0.226	8.129	3.027***		
Family	-0.151***	[-0.201, -0.101]	0.025	-5.941	-0.324***		
Friends	-0.022	[-0.088, 0.044]	0.034	-0.649	-0.039		
Significant Other	-0.035	[-0.094, 0.025]	0.03	-1.151	-0.07		
Network Size	0.018	[-0.008, 0.044]	0.013	1.357	0.074		
Quality of Ties	-0.197**	[-0.346, -0.048]	0.076	-2.588	-0.196**		
Closeness of Ties	0.233***	[0.079, 0.388]	0.079	2.971	0.232***		

** *p* <.01, *** *p* <.001. *N* = 412. PSS = Perceived Social Support.

### Model 2 - Main effects of social networks

In Model 2, we estimated the main associations between individuals’ social network characteristics and mental distress. Among quality, closeness, and size of social networks, only the quality of networks showed a statistically significant relationship with mental distress (β = -.288, *p* < .001). The association between the quality of networks and mental distress was negative, meaning that increasing quality of networks is a negative predictor for mental distress. Model 2 explained 4.7% of variance in mental distress (*R*^*2*^ *=* .047).

### Model 3 - Main effects of both perceived social support and social networks

Model 3 combined perceived social support and the characteristics of social networks to investigate the main associations between social support, network characteristics, and mental distress. Among the different components of social support, the estimated coefficient for family support was again negative and significantly associated with mental distress (β = -.324, *p* < .001). In terms of social networks, the quality of networks was again negative and significantly associated with mental distress (β = -.196, *p* = .01). In addition, network closeness unexpectedly showed a significant and positive association with mental distress (β = .232, *p* = .003), suggesting that students who felt closer to their network members were associated with higher levels of mental distress. Model 3 explained 16% of variance in mental distress (*R*^*2*^ *=* .16).

### Robustness check using graduate student status

In addition to the main models described above, we also tested an alternate set of models which were identical but for the addition of a predictor of whether the student was a graduate student or an undergraduate. Given that graduate students are more likely to live independently year round, and tend to be older than undergraduates, student status was a potential confounder of the relationships we tested. S1 Table in the supplementary materials (S1 Table) includes regression results for all three models with the additional graduate student predictor. The strength, direction, and significance of the variables identified above did not substantively change once controlling for graduate student status.

## Discussion

The preceding analysis demonstrated the importance of family support in mitigating college students’ mental distress during the COVID-19 lockdown, and also illustrated the importance of including social network characteristics when examining the relationship between social support and mental health in this context. These findings extend the current literature on perceived social support, social network ties, and mental health of undergraduate students in several ways.

### Perceived social support

The results of our models suggest that family support served an important role regarding college students’ mental distress during the COVID-19 lockdown. Due to the lockdown and campus closure at the time of our survey, students were mostly socially isolated and were required to stay at home. Hence they may have relied more on family for social support than in a typical college setting. This finding confirms findings from Wesley and Booker’s study [22], which showed that family support was consistently associated with well-being, stress and depression, whereas friend support was only associated with well-being and stress during the COVID-19 disruptions in Spring 2020. Both of our results suggest that family support is a pivotal factor in the relationship between perceived social support and mental distress, especially when dealing with isolated scenarios like pandemic lockdown. However, friends’ support was not significantly associated with mental distress in our study.

### Social network ties

Adding characteristics of individuals’ social networks, such as network size, quality, and closeness of network ties, into our model significantly increased the explained variance of mental distress. Among the social network characteristics we included, quality of networks was a consistent negative predictor of mental distress, both by itself and when introduced alongside perceived social support. This finding is similar to past research investigating relations between social ties (i.e., loneliness, isolation) and mental health [38,51]. In addition, our results showed that closeness with network members was positively associated with mental distress. This finding is partly conflicted with previous research during COVID-19 in South Korea [52], which found that level of closeness with networks was negatively associated with PTSD score among women. We suspect that those who reported higher closeness with their social network members might perceive much higher anxiety and depression in the extremely isolated lockdown scenario, where those close network members may not be readily accessible. An alternative explanation of the negative association between closeness and mental distress is that emotional contagion was occurring between family members during lockdown. Prior research has shown that especially in relatively isolated social networks, such as one might find during lockdown, negative mood is more contagious than positive mood [53]. In addition, measurement limitations may have played a role in these findings, as our measurement of closeness was only a single item measured on a Likert-type scale. A more robust measure with multiple items might improve the precision of these scores and offer more clear results.

### Integration of networks and social support

Having discussed the individual contributions of the paper to the fields of social networks (the role of quality of ties) and social support research (the role of family support), we also believe that this paper demonstrates how these two concepts overlap. For example, 49.0% of all ties identified were from family (parent, sibling, other family member), while slightly fewer (34.7%) were from friends. This aligns with the context, in which most students were in lockdown at home and had the easiest access to family members. This underscores the point that quality family-based ties may have played an especially important role during this critical time period.

This reveals the importance of incorporating social networks when investigating the relationship between perceived social support on mental health in a college setting. Our approach of asking respondents to list specific network members aligns with the concept of rating specific support providers, as suggested by Lakey and Orehek [11]. Adding social network information allows researchers to better measure the regulation of emotion via relationships when investigating the main effect of social support on mental health.

## Limitations and future directions

This work has a number of limitations that should be enumerated, and also opens up a number of future directions for research. First, we did not attempt to make any causal inferences about the relationship between the social support, network properties, and the outcomes we measured in the present study. This dataset represents students’ perspectives at a single, unique point in time at the beginning of the pandemic, and we did not introduce any randomized element to the study that might facilitate a causal claim. The findings we reported here are associational and not causal. Other unmeasured variables could have an impact on these relationships and potentially shift our interpretation. Indeed, given the relatively small amount of variance explained by our models (between 4 and 16%), there are likely many other additional variables that could be considered to explain more variation in mental distress scores. For example, a measure of Covid exposure or diagnosis, as well as a measure of specific stress due to the pandemic (e.g., providing care to sick friends or family) might offer additional nuance to our findings. Further, given the cross-sectional nature of the data collection, we are unable to rule out a reversed relationship between distress and social support (i.e., the presence of increased mental distress could potentially heighten perceptions of family support or closeness). Longitudinal studies in the future could help tease out this causal structure more explicitly.

Given that our study uses data from a single site, we also do not claim that our findings would generalize to all universities, or even all university settings in the US. Our sample was collected from a large, public university in the Mid-Atlantic US and was composed of a majority of female students and primarily undergraduates. To the extent that other university populations might differ from this profile, we would expect that results from a similar investigation may differ.

Finally, given the findings of our study, some future directions for research have emerged. First, we encourage future research to include social network ties when discussing perceived social support. Not only because in our results, social network ties improved the predictive power of all models, but also that it is reasonable to include social network analysis under the theoretical framework of social capital. Last, we agree with the point of view from RRT that research on perceived social support should consider asking respondents to name specific support providers in order to obtain more accurate estimation of perceived social support. The name generators in social network analysis could be used to fulfill this requirement [39], which is the approach we presented in this study.

## Recommendations for policy and practice

This work also highlights several recommendations for policy and practice in higher education and student affairs. First, building strong friendship networks in college is crucial for supporting students’ mental health, and colleges should implement policies that encourage the development of these networks. Our findings indicate that while online interactions have grown increasingly prevalent, they do not exert the same influence on students as in-person connections. Instead, deliberate, institution-supported initiatives that promote face-to-face interactions can be more effective in fostering robust, quality ties. This suggests that schools should prioritize everyday interactions, such as simple, regular social encounters that build trust and familiarity, over relying solely on digital platforms to generate meaningful relationships.

Further, to mitigate social isolation, it is important that schools create environments where consistent and durable social connections can flourish. For example, altering campus layouts to create inviting common spaces, designing orientation programs that include diverse and interactive social activities, and supporting faculty-led initiatives that integrate social components into academic experiences can provide multiple avenues for students to build and maintain close ties.

Finally, the intentional development of friendship networks as a component of student well-being can be encouraged by embedding these principles into everyday campus life. By routinely engaging in small-group activities and communal events, students are more likely to form lasting connections that extend beyond brief encounters. Administrators and policymakers should adopt evidence-based strategies that target social isolation and reinforce the mental health benefits of sustained social support. These strategies might include routine assessments of student social engagement and well-being, the implementation of peer mentoring programs, and the creation of digital platforms designed to supplement in-person interactions without replacing them.

## Conclusion

In summary, our study makes a significant contribution to the emerging research on the relationships between college students’ mental health, perceived social support, and social networks during the onset of the COVID-19 pandemic. Without question, this unusual and singular period has had a dramatic impact on postsecondary education, and perhaps more importantly, postsecondary students. Our hope is that this work might help future researchers make more sense of the complexity of social relationships during this time in an effort to ameliorate potential negative effects on mental health for students.

## Supporting information

S1 Table Path Analysis Results, Controlling for Graduate Student Status. (DOCX)

## References

[1] American Educational Research Association, Levine F, Nasir NS, Rios-Aguilar C, Gildersleeve R, Rosich K, et al. Voices from the field: The impact of COVID-19 on early career scholars and doctoral students [Internet]. American Educational Research Association; 2021 Mar [cited 2024 Sep 2]. Available from: https://www.aera.net/Education-Research/Voices-from-the-Field-The-Impact-of-COVID-19-on-Early-Career-Scholars-and-Doctoral-Students

[2] Govindarajan V, Srivastava A. A Post-Pandemic Strategy for U.S. Higher Ed. Harvard Business Review [Internet]. 2020 Jun 2 [cited 2021 Jun 2]; Available from: https://hbr.org/2020/06/a-post-pandemic-strategy-for-u-s-higher-ed

[3] Toquero CM. Challenges and Opportunities for Higher Education amid the COVID-19 Pandemic: The Philippine Context. PEDAGOGICAL RES. 2020;5(4):em0063. doi: 10.29333/pr/7947

[4] Bayram N, Bilgel N. The prevalence and socio-demographic correlations of depression, anxiety and stress among a group of university students. Soc Psychiatry Psychiatr Epidemiol. 2008;43(8):667–72. doi: 10.1007/s00127-008-0345-x 18398558

[5] Beiter R, Nash R, McCrady M, Rhoades D, Linscomb M, Clarahan M, et al. The prevalence and correlates of depression, anxiety, and stress in a sample of college students. J Affect Disord. 2015;173:90–6. doi: 10.1016/j.jad.2014.10.054 25462401

[6] Mahmoud JSR, Staten R, Hall LA, Lennie TA. The relationship among young adult college students’ depression, anxiety, stress, demographics, life satisfaction, and coping styles. Issues in Mental Health Nursing. 2012;33(3):149–56. 22364426 10.3109/01612840.2011.632708

[7] Kecojevic A, Basch CH, Sullivan M, Davi NK. The impact of the COVID-19 epidemic on mental health of undergraduate students in New Jersey, cross-sectional study. PLoS One. 2020;15(9):e0239696. doi: 10.1371/journal.pone.0239696 32997683 PMC7526896

[8] Son C, Hegde S, Smith A, Wang X, Sasangohar F. Effects of COVID-19 on College Students’ Mental Health in the United States: Interview Survey Study. J Med Internet Res. 2020;22(9):e21279. doi: 10.2196/21279 32805704 PMC7473764

[9] Bridgland VME, Moeck EK, Green DM, Swain TL, Nayda DM, Matson LA, et al. Why the COVID-19 pandemic is a traumatic stressor. PLoS One. 2021;16(1):e0240146. doi: 10.1371/journal.pone.0240146 33428630 PMC7799777

[10] Griffin G. Defining trauma and a trauma-informed COVID-19 response. Psychological Trauma: Theory, Research, Practice, and Policy. 2020;12(S1):S279-80. 10.1037/tra0000828 32551754

[11] Lakey B, Orehek E. Relational regulation theory: a new approach to explain the link between perceived social support and mental health. Psychol Rev. 2011;118(3):482–95. doi: 10.1037/a0023477 21534704

[12] McKenzie K, Harpham T, editors. Social capital and mental health. London, England: Jessica Kingsley Publishers; 2006. 160 p. (Social capital and mental health).

[13] Robison LJ, Siles ME, Schmid AA. Social capital and poverty reduction: toward a mature paradigm. Agricultural Economics Report No 614 [Internet]. 2002 [cited 2024 Sep 2]; Available from: https://ageconsearch.umn.edu/record/10941

[14] Bourdieu P. The Force of Law: Toward a Sociology of the Juridical Field. Hastings Law Journal. 1986;38:805.

[15] Coleman JS. Social Capital in the Creation of Human Capital. American Journal of Sociology [Internet]. 1988 [cited 2024 Sep 2];94. Available from: https://www.journals.uchicago.edu/doi/abs/10.1086/228943

[16] Putnam R. Social capital: Measurement and consequences. Canadian journal of policy research. 2001;2(1):41–51.

[17] Almedom AM. Social capital and mental health: an interdisciplinary review of primary evidence. Soc Sci Med. 2005;61(5):943–64. doi: 10.1016/j.socscimed.2004.12.025 15955397

[18] Putnam RD. Bowling Alone: The Collapse and Revival of American Community. Simon and Schuster. 2000.

[19] Mitchell CU, LaGory M. Social capital and mental distress in an impoverished community. City & Community. 2002;1(2):199.

[20] Forsman AK, Nyqvist F, Schierenbeck I, Gustafson Y, Wahlbeck K. Structural and cognitive social capital and depression among older adults in two Nordic regions. Aging Ment Health. 2012;16(6):771–9. doi: 10.1080/13607863.2012.667784 22486561

[21] Lin N. Social networks and status attainment. Annual Review of Sociology. 1999;25(1):467–87.

[22] Wesley R, Booker JA. Social Support and Psychological Adjustment Among College Adults. Journal of Social and Clinical Psychology. 2021;40(1):69–95. doi: 10.1521/jscp.2021.40.1.69

[23] Brandy JM, Penckofer S, Solari-Twadell PA, Velsor-Friedrich B. Factors predictive of depression in first-year college students. J Psychosoc Nurs Ment Health Serv. 2015;53(2):38–44. doi: 10.3928/02793695-20150126-03 25654575

[24] Zimet GD, Dahlem NW, Zimet SG, Farley KG, Hollingsworth DW, Slish ML. Multidimensional Scale of Perceived Social Support: The Indirect Effect of Perceived Burdensomeness on the Relationship Between Indices of Social Support and Suicide Ideation in College Students. Journal of American College Health. 2018;66(1):9–16. 28812441 10.1080/07448481.2017.1363764

[25] Rueger SY, Malecki CK, Demaray MK. Relationship between multiple sources of perceived social support and psychological and academic adjustment in early adolescence: Comparisons across gender. J Youth Adolescence. 2010;39(1):47–61. 10.1007/s10964-008-9368-6 20091216

[26] Swift A, Wright MO. Does Social Support Buffer Stress for College Women. Journal of College Student Psychotherapy. 2000;14(4):23–42. doi: 10.1300/j035v14n04_05

[27] DeBerard MS, Spielmans GI, Julka DL. Predictors of academic achievement and retention among college freshmen: a longitudinal study. College Student Journal. 2004;38(1):66–81.

[28] Robbins SB, Lauver K, Le H, Davis D, Langley R, Carlstrom A. Do psychosocial and study skill factors predict college outcomes? A meta-analysis. Psychol Bull. 2004;130(2):261–88. doi: 10.1037/0033-2909.130.2.261 14979772

[29] Rawson HE, Bloomer K, Kendall A. Stress, anxiety, depression, and physical illness in college students. J Genet Psychol. 1994;155(3):321–30. doi: 10.1080/00221325.1994.9914782 7964658

[30] Yasin MASM, Dzulkifli MA. The Relationship between Social Support and Psychological Problems among Students. 2010. Available from: http://umt-ir.umt.edu.my:8080/jspui/handle/123456789/4230

[31] Mauer VA, Littleton H, Lim S, Sall KE, Siller L, Edwards KM. Fear of COVID-19, anxiety, and social support among college students. J Am Coll Health. 2024;72(2):631–8. doi: 10.1080/07448481.2022.2053689 35325590

[32] Reid C, Beckstead J, Salinas-Miranda A. COVID-19 stress, social support, and coping in international students during the COVID-19 pandemic: a moderated analysis on anxiety and depression. J Am Coll Health. 2024;72(5):1617–23. doi: 10.1080/07448481.2022.2089044 35728102

[33] De Silva MJ, McKenzie K, Harpham T, Huttly SRA. Social capital and mental illness: a systematic review. J Epidemiol Community Health. 2005;59(8):619–27. doi: 10.1136/jech.2004.029678 16020636 PMC1733100

[34] Eggens L, van der Werf MPC, Bosker RJ. The influence of personal networks and social support on study attainment of students in university education. High Educ. 2008;55(5):553–73.

[35] Hurd NM, Albright J, Wittrup A, Negrete A, Billingsley J. Appraisal support from natural mentors, self-worth, and psychological distress: Examining the experiences of underrepresented students transitioning through college. Journal of Youth and Adolescence. 2018;47(5):1100–12. 29282606 10.1007/s10964-017-0798-x

[36] Mason MJ, Zaharakis N, Benotsch EG. Social Networks, Substance Use, and Mental Health in College Students. Journal of American College Health. 2014;62(7):470–7. 24848433 10.1080/07448481.2014.923428

[37] Taylor RJ, Chae DH, Lincoln KD, Chatters LM. Extended family and friendship support networks are both protective and risk factors for major depressive disorder and depressive symptoms among African-Americans and black Caribbeans. J Nerv Ment Dis. 2015;203(2):132–40. doi: 10.1097/NMD.0000000000000249 25594791 PMC4310769

[38] Elmer T, Mepham K, Stadtfeld C. Students under lockdown: Comparisons of students’ social networks and mental health before and during the COVID-19 crisis in Switzerland. PLoS One. 2020;15(7):e0236337. doi: 10.1371/journal.pone.0236337 32702065 PMC7377438

[39] Broda MD, Granger K, Chow J, Ross E. Using social network analysis in applied psychological research: A tutorial. Psychol Methods. 2023;28(4):791–805. doi: 10.1037/met0000451 34914476

[40] Wasserman S, Faust K. Social Network Analysis: Methods and Applications [Internet]. 1st ed. Cambridge University Press; 1994 [cited 2024 Sep 9]. Available from: https://www.cambridge.org/core/product/identifier/9780511815478/type/book

[41] Dahlem NW, Zimet GD, Walker RR. The Multidimensional Scale of Perceived Social Support: a confirmation study. J Clin Psychol. 1991;47(6):756–61. doi: 10.1002/1097-4679(199111)47:6<756::aid-jclp2270470605>3.0.co;2-l 1757578

[42] Kazarian SS, McCabe SB. Dimensions of social support in the MSPSS: Factorial structure, reliability, and theoretical implications. J Community Psychol. 1991;19(2):150–60. doi: 10.1002/1520-6629(199104)19:2<150::aid-jcop2290190206>3.0.co;2-j

[43] Beaufort IN, De Weert-Van Oene GH, Buwalda VAJ, de Leeuw JRJ, Goudriaan AE. The Depression, Anxiety and Stress Scale (DASS-21) as a Screener for Depression in Substance Use Disorder Inpatients: A Pilot Study. Eur Addict Res. 2017;23(5):260–8. doi: 10.1159/000485182 29224000

[44] Lovibond PF, Lovibond SH. The structure of negative emotional states: comparison of the Depression Anxiety Stress Scales (DASS) with the Beck Depression and Anxiety Inventories. Behav Res Ther. 1995;33(3):335–43. doi: 10.1016/0005-7967(94)00075-u 7726811

[45] Osman A, Wong JL, Bagge CL, Freedenthal S, Gutierrez PM, Lozano G. The Depression Anxiety Stress Scales—21 (DASS‐21): Further Examination of Dimensions, Scale Reliability, and Correlates. J Clin Psychol. 2012;68(12):1322–38. 22930477 10.1002/jclp.21908

[46] Coromina L, Coenders G. Reliability and validity of egocentered network data collected via web. Social Networks. 2006;28(3):209–31. doi: 10.1016/j.socnet.2005.07.006

[47] DeVellis RF, Thorpe CT. Scale Development: Theory and Applications. SAGE Publications. 2021.

[48] R Core Team. R: A Language and Environment for Statistical Computing. 2024.

[49] Rosseel Y. lavaan: An *R* Package for Structural Equation Modeling. J Stat Soft [Internet]. 2012 [cited 2024 Sep 9];48(2). Available from: http://www.jstatsoft.org/v48/i02/

[50] Cohen J. Statistical power analysis for the behavioral sciences. 2nd ed. New York: Routledge. 1988.

[51] Levula A, Harré M. Social networks and mental health: an egocentric perspective. MHRJ. 2016;21(3):161–73. doi: 10.1108/mhrj-10-2015-0029

[52] Yang JS, Lee YJ, Kim HC, Cho C-H, Tsai AC, Jung SJ. Association between social networks and symptoms of post-traumatic stress during the pandemic: Cohort study in South Korea. Compr Psychiatry. 2023;127:152432. doi: 10.1016/j.comppsych.2023.152432 37856975

[53] Block P, Burnett Heyes S. Sharing the load: Contagion and tolerance of mood in social networks. Emotion. 2022;22(6):1193–207. doi: 10.1037/emo0000952 33370141

