# Supplementary material for: Exploring relationships between college students’ social networks, social support, and mental health during the COVID-19 pandemic
Source: PLOS Ment Health. 2026 Mar 20;3(3):e0000289. doi: 10.1371/journal.pmen.0000289 (PMC13004404; doi:10.1371/journal.pmen.0000289)
Supplement: S1 Table — (DOCX) [file pmen.0000289.s001.docx]

Table S1.

*Path Analysis Results, Controlling for Graduate Student Status*

| Predictors | *B* | *SE* | *Z* | β |
| --- | --- | --- | --- | --- |
| Model 1 - PSS |  |  |  |  |
| (Intercept) | 1.764*** | 0.153 | 11.511 | 2.907*** |
| Family | -0.153*** | 0.025 | -6.069 | -0.328*** |
| Friends | -0.002 | 0.033 | -0.063 | -0.004 |
| Significant Other | -0.041 | 0.03 | -1.368 | -0.083 |
| Graduate Student (Yes) | 0.055 | 0.068 | 0.810 | 0.039 |
| Model 2 - Network Ties |  |  |  |  |
| (Intercept) | 1.516*** | 0.238 | 6.378 | 2.499*** |
| Network Size | 0.018 | 0.015 | 1.202 | 0.072 |
| Quality of Ties | -0.277*** | 0.080 | -3.450 | -0.276*** |
| Closeness of Ties | 0.125 | 0.081 | 1.541 | 0.122 |
| Graduate Student (Yes) | -0.049 | 0.070 | -0.694 | -0.034 |
| Model 3 - Combined Model |  |  |  |  |
| (Intercept) | 1.853*** | 0.226 | 8.208 | 3.056*** |
| Family | -0.160*** | 0.026 | -6.112 | -0.343*** |
| Friends | -0.021 | 0.034 | -0.633 | -0.038 |
| Significant Other | -0.038 | 0.030 | -1.265 | -0.077 |
| Network Size | 0.019 | 0.013 | 1.429 | 0.078 |
| Quality of Ties | -0.205** | 0.069 | -2.683 | -0.205** |
| Closeness of Ties | 0.247** | 0.080 | 3.112 | 0.246** |
| Graduate Student (Yes) | 0.097 | 0.069 | 1.405 | 0.068 |

** *p* < .01, *** *p* < .001. N = 412. PSS = Perceived Social Support.
